# Checklist of the family Sphaeroceridae (Diptera) of Finland

**DOI:** 10.3897/zookeys.441.7250

**Published:** 2014-09-19

**Authors:** Antti Haarto, Jere Kahanpää

**Affiliations:** 1 Zoological Museum, Section of Biodiversity and Environmental Science, Department of Biology, FI-20014 University of Turku, Finland; 2 Finnish Museum of Natural History, Zoology Unit, P.O. Box 17, FI–00014 University of Helsinki, Finland

**Keywords:** Checklist, Finland, Diptera, Sphaeroceridae

## Abstract

A checklist of the family Sphaeroceridae (Diptera) recorded from Finland.

## Introduction

Sphaeroceridae or lesser dung flies are mostly small and dull dark brown to grey acalyptrate flies. They are easily distinguished from other acalyptratae because of the short and thickened basal tarsomeres of hind legs. Larvae of Sphaeroceridae develop in decaying organic matter such as decaying vegetation, dung and rotten fungi.

The family has gathered little interest among entomologists in Finland. Walter Hackman concentrated on the taxonomy of the subfamily Copromyzinae (Hackman 1965) and investigated the dipterous fauna, including sphaerocerids, in the burrows of small mammals (Hackman 1963). Hackman (1980) published the latest list of the Finnish sphaerocerid species. Further species were added in several revisions of European taxa by Roháček (1982, 1983, 1991). There has been increasing interest in sphaerocerids in Finland after the year 2000 (see Haarto and Kahanpää 2013).

The classification follows the world catalogue of Sphaeroceridae (Roháček et al. 2001, Marshall et al. 2011).

**Number of species:**

World: 1571 species (Pape et al. 2011)

Europe: 257 species

Finland: 118 species

Faunistic knowledge level in Finland: average

## Checklist

suborder Brachycera Macquart, 1834

clade Eremoneura Lameere, 1906

clade Cyclorrhapha Brauer, 1863

infraorder Schizophora Becher, 1882

clade Muscaria Enderlein, 1936

parvorder Acalyptratae Macquart, 1835

superfamily Sphaeroceroidea Macquart, 1835

**SPHAEROCERIDAE** Macquart, 1835

COPROMYZINAE Stenhammar, 1854

***ALLOBORBORUS*** Duda, 1923

*Alloborborus pallifrons* (Fallén, 1820)

= *flavipennis* (Haliday, 1836)

***BORBORILLUS*** Duda, 1923

*Borborillus uncinatus* (Duda, 1923)

*Borborillus vitripennis* (Meigen, 1830)

= *longipennis* (Haliday, 1836)

***COPROMYZA*** Fallén, 1810

*Copromyza borealis* Zetterstedt, 1847

*Copromyza equina* Fallén, 1820

*Copromyza nigrina* (Gimmerthal, 1847)

= *similis* (Collin, 1930)

*Copromyza stercoraria* (Meigen, 1830)

***CRUMOMYIA*** Macquart, 1835

= ***Apterina*** Macquart, 1835

*Crumomyia fimetaria* (Meigen, 1830)

*Crumomyia gelida* Hackman, 1965

*Crumomyia glabrifrons* (Meigen, 1830)

*Crumomyia nigra* (Meigen, 1830)

*Crumomyia nitida* (Meigen, 1830)

*Crumomyia notabilis* (Collin, 1902)

= *glacialis* misid.

*Crumomyia pedestris* (Meigen, 1830

*Crumomyia pruinosa* (Richards, 1932)

= *annulus* misid.

= *rufoannulata* misid.

*Crumomyia setitibialis* (Spuler, 1925)

= *freyi* (Hackman, 1965)

= *annulipes* misid.

= *roseri* misid.

***LOTOPHILA*** Lioy, 1864

*Lotophila atra* (Meigen, 1830)

***NORRBOMIA*** Papp, 1988

*Norrbomia costalis* (Zetterstedt, 1847)

*Norrbomia fumipennis* (Stenhammar, 1855)

*Norrbomia hispanica* (Duda, 1923)

*Norrbomia sordida* (Zetterstedt, 1847)

SPHAEROCERINAE Macquart, 1835

***ISCHIOLEPTA*** Lioy, 1864

*Ischiolepta crenata* (Meigen, 1838)

*Ischiolepta denticulata* (Meigen, 1830)

= *paracrenata* (Duda, 1920)

*Ischiolepta micropyga* (Duda, 1938)

*Ischiolepta nitida* (Duda, 1920)

= *denticulata* auct. nec (Meigen, 1830)

*Ischiolepta pusilla* (Fallén, 1820)

*Ischiolepta scabricula* (Haliday, 1836)

*Ischiolepta vaporariorum* (Haliday, 1836)

***LOTOBIA*** Lioy, 1864

*Lotobia pallidiventris* (Meigen, 1830)

***SPHAEROCERA*** Latreille, 1804

= ***Cypsela*** Meigen, 1800 suppr.

*Sphaerocera curvipes* Latreille, 1805

*Sphaerocera monilis* Haliday, 1836

LIMOSININAE Frey, 1921

***APTEROMYIA*** Vimmer, 1929

*Apteromyia claviventris* (Strobl, 1909)

***BIFRONSINA*** Roháček, 1983

*Bifronsina bifrons* (Stenhammar, 1855)

***CHAETOPODELLA*** Duda, 1920

**sg. *Chaetopodella*** Duda, 1920

*Chaetopodella scutellaris* (Haliday, 1836)

***COPROICA*** Rondani, 1861

*Coproica acutangula* (Zetterstedt, 1847)

*Coproica ferruginata* (Stenhammar, 1855)

*Coproica hirticula* Collin, 1965

*Coproica hirtula* (Rondani, 1880)

*Coproica lugubris* (Haliday, 1835)

*Coproica pusio* (Zetterstedt, 1847)

= *pseudolugubris* (Duda, 1924)

*Coproica vagans* (Haliday, 1833)

***ELACHISOMA*** Rondani, 1880

*Elachisoma aterrimum* (Haliday, 1833)

***EULIMOSINA*** Roháček, 1983

*Eulimosina ochripes* (Meigen, 1830)

***GIGALIMOSINA*** Roháček, 1983

*Gigalimosina flaviceps* Zetterstedt, 1847

***GONIONEURA*** Rondani, 1880

= ***Halidayina*** Duda, 1918

*Gonioneura spinipennis* (Haliday, 1836)

***HERNIOSINA*** Roháček, 1983

*Herniosina bequaerti* (Villeneuve, 1917)

***LEPTOCERA*** Olivier, 1813

= ***Paracollinella*** Duda, 1924

*Leptocera caenosa* (Rondani, 1880)

*Leptocera finalis* (Collin, 1956)

*Leptocera fontinalis* (Fallén, 1826)

*Leptocera nigra* Olivier, 1813

***LIMOSINA*** Macquart, 1835

*Limosina silvatica* (Meigen, 1830)

***MINILIMOSINA*** Roháček, 1983

**sg. *Minilimosina*** Roháček, 1983

*Minilimosina baculum* Marshall, 1985

*Minilimosina bicuspis* Roháček, 1993

= *trogeri* misid.

*Minilimosina fungicola* (Haliday, 1836)

= *exigua* (Rondani, 1880)

*Minilimosina parvula* Stenhammar, 1855

*Minilimosina tenera* Roháček, 1983

**sg. *Svarciella*** Roháček, 1983

*Minilimosina guestphalica* (Duda, 1918)

= *v-atrum* misid.

*Minilimosina v-atrum* (Villeneuve, 1917)

= *splendens* (Duda, 1928)

*Minilimosina vitripennis* (Zetterstedt, 1847)

***OPACIFRONS*** Duda, 1918

*Opacifrons coxata* (Stenhammar, 1855)

***OPALIMOSINA*** Roháček, 1983

**sg. *Dentilimosina*** Roháček, 1983

*Opalimosina denticulata* (Duda, 1924)

**sg. *Opalimosina*** Roháček, 1983

*Opalimosina collini* (Richards, 1929)

*Opalimosina mirabilis* (Collin, 1902)

*Opalimosina simplex* Richards 1929

**sg. *Pappiella*** Roháček, 1983

*Opalimosina liliputana* (Rondani, 1880)

= *appendiculata* Villeneuve, 1918

***PARALIMOSINA*** Papp, 1973

*Paralimosina kaszabi* Papp, 1973

***PHILOCOPRELLA*** Richards, 1929

*Philocoprella quadrispina* (Laurence, 1952)

***PHTHITIA*** Enderlein, 1938

**sg. *Alimosina*** Roháček, 1983

*Phthitia empirica* (Hutton, 1901)

= *cadaverina* (Duda, 1918)

**sg. *Collimosina*** Roháček, 1983

*Phthitia spinosa* Collin, 1930

**sg. *Kimosina*** Roháček, 1983

*Phthitia longisetosa* (Dahl, 1909)

*Phthitia plumosula* (Rondani, 1880)

***PSEUDOCOLLINELLA*** Duda, 1924

*Pseudocollinella flavilabris* (Hackman, 1968)

*Pseudocollinella humida* (Haliday, 1836)

*Pseudocollinella septentrionalis* (Stenhammar, 1855)

***PTEREMIS*** Rondani, 1856

*Pteremis fenestralis* (Fallén, 1820)

= *nivalis* (Haliday, 1833)

= *subaptera* Frey, 1946

***PULLIMOSINA*** Roháček, 1983

**sg. *Dahlimosina*** Roháček, 1983

*Pullimosina dahli* (Duda, 1918)

**sg. *Pullimosina*** Roháček, 1983

*Pullimosina heteroneura* (Haliday, 1836)

*Pullimosina meijerei* (Duda, 1918)

*Pullimosina moesta* (Villeneuve, 1918)

= *antennata* (Duda, 1918)

*Pullimosina pullula* (Zetterstedt, 1847)

*Pullimosina vulgesta* Roháček, 2001

= *moesta* auct. nec (Villeneuve, 1918)

***RACHISPODA*** Lioy, 1864

*Rachispoda anceps* (Stenhammar, 1855)

*Rachispoda breviceps* (Stenhammar, 1855)

*Rachispoda fuscipennis* (Haliday, 1833)

*Rachispoda hostica* Villeneuve, 1917

*Rachispoda intermedia* (Duda, 1918)

*Rachispoda limosa* (Zetterstedt, 1820)

*Rachispoda lugubrina* (Zetterstedt, 1847)

*Rachispoda lutosa* (Stenhammar, 1855)

= *palustris* (Collin, 1930)

*Rachispoda lutosoidea* (Duda, 1938)

= *lutosa* auct. nec (Stenhammar, 1855)

***SPELOBIA*** Spuler, 1924

*Spelobia belanica* Roháček, 1983

*Spelobia cambrica* (Richards, 1929)

*Spelobia clunipes* (Meigen, 1830)

= *crassimana* (Haliday, 1836)

*Spelobia ibrida* Roháček, 1983

*Spelobia luteilabris* (Rondani, 1880)

*Spelobia manicata* (Richards, 1927)

*Spelobia nana* (Rondani, 1880)

*Spelobia palmata* (Richards, 1927)

*Spelobia pappi* Roháček, 1983

*Spelobia parapusio* (Dahl, 1909)

*Spelobia pseudonivalis* (Dahl, 1909)

*Spelobia pseudosetaria* (Duda, 1918)

= *penetralis* (Collin, 1925)

*Spelobia rufilabris* (Stenhammar, 1855)

*Spelobia talparum* (Richards, 1927)

*Spelobia ulla* Roháček, 1983

***SPINILIMOSINA*** Roháček, 1983

*Spinilimosina brevicostata* (Duda, 1918)

***TELOMERINA*** Roháček, 1983

*Telomerina eburnea* Roháček, 1983

*Telomerina flavipes* (Meigen, 1830)

*Telomerina pseudoleucoptera* (Duda, 1924)

***TERRILIMOSINA*** Roháček, 1983

*Terrilimosina racovitzai* (Bezzi, 1911)

*Terrilimosina schmitzi* (Duda, 1918)

***THORACOCHAETA*** Duda, 1918

*Thoracochaeta brachystoma* (Stenhammar, 1855)

*Thoracochaeta zosterae* (Haliday, 1833)

***TRACHYOPELLA*** Duda, 1918

**sg. *Nudopella*** Roháček & Marshall, 1986

*Trachyopella leucoptera* (Haliday, 1836)

**sg. *Trachyopella*** Duda, 1918

*Trachyopella atomus* (Rondani, 1880)

*Trachyopella bovilla* Collin, 1954

= *coprina* misid.

*Trachyopella lineafrons* (Spuler, 1925)

= *atomus* misid.

*Trachyopella melania* (Haliday, 1836)

= *villeneuvii* (Duda, 1924)

## Excluded species

*Rachispoda cilifera* (Rondani, 1880) not found within present borders

*Minilimosina unica* (Papp, 1973) not found within present borders

= *hackmani* (Roháček, 1977)

## Notes

***Spelobia manicata* (Richards, 1927)** is considered a valid species although Pitkin (1988) synonymized it with *Spelobia clunipes* (Meigen, 1830).

## References

[B1] Haarto A Kahanpää J (2013) Notes of Finnish Sphaeroceridae (Diptera) with description of the female of *Minilimosina tenera* Rohacek, 1983. Entomologica Fennica 24: 228–233. http://ojs.tsv.fi/index.php/entomolfennica/article/download/9385/6695

[B2] Hackman W (1963) Studies on the dipterous fauna in burrows of voles (*Microtus*, *Clethrionomys*) in Finland. Acta Zoologica Fennica 102: 1–64

[B3] Hackman W (1965) On the genus Copromyza Fall. (Dipt., Sphaeroceridae), with special reference to the Finnish species. Notulae Entomologicae 45: 33–46

[B4] Hackman W (1980) A Check List of the Finnish Diptera. Notulae entomologicae 60: 17–48, 117–162

[B5] Marshall SA Roháček J Dong H Buck M (2011) The state of Sphaeroceridae (Diptera: Acalyptratae): a world catalog update covering the years 2000-2010, with new generic synonymy, new combinations, and new distributions. Acta Entomologica Musei Nationalis Pragae 51(1): 217–298. http://www.aemnp.eu/PDF/51_1/51_1_217.pdf

[B6] Pape T Blagoderov V Mostovski MB (2011) Order Diptera Linnaeus, 1758. In: Zhang Z-Q (Ed) Animal biodiversity: An outline of higher-level classification and survey of taxonomic richness.Zootaxa 3148: 222–229. http://www.mapress.com/zootaxa/2011/f/zt03148p229.pdf 10.11646/zootaxa.3703.1.1 26146682

[B7] Pitkin BR (1988) Lesser dung flies, Diptera: Sphaeroceridae. Handbooks for the Identification of British Insects, Vol 10, Part 5e. Royal Entomological Society of London, London, 175 pp

[B8] Roháček J (1982) Revision of the subgenus *Leptocera* (s.str.) of Europe. Entomologische Abhandlungen Staatliches Museum für Tierkunde in Dresden 46(1): 1–44

[B9] Roháček J (1983) A monograph and re-classification of the previous genus *Limosina* Macquart (Diptera, Sphaeroceridae) of Europe. Part II. Beiträge zur Entomologie, Berlin 33: 3–195

[B10] Roháček J (1991) A monograph of Leptocera (Rachispoda Lioy) of the West Palaearctic area (Diptera, Sphaeroceridae). Časopis Slezského zemského Muzea, Opava (A) 40: 97–288

[B11] Roháček J Marshall SA Norrbom AL Buck M Quiros DI Smith I (2001) World Catalog of Sphaeroceridae. Slezské Zemské Muzeum, Opava, 300 pp. ISBN 808622421X. http://www.uoguelph.ca/debu/catalog.htm

